# Effects of two versus three weekly sessions of low-load elastic band resistance training on functional fitness and metabolic biomarkers in older adults: a randomized controlled trial

**DOI:** 10.1186/s11556-026-00427-9

**Published:** 2026-07-13

**Authors:** Tatjana Jezdimirović Stojanović, Nikola Andrić, Natasa Zenić, Ane Rincon Perez, Marko D.M. Stojanović

**Affiliations:** 1 Faculty of Sport, Union Nikola Tesla University, Belgrade, Serbia and Montenegro; 2 Training expertise laboratory, Novi Sad, Serbia and Montenegro; 3 Faculty of Sport and Physical Education, University of Novi Sad, Novi Sad, Serbia and Montenegro; 4 Faculty of Kinesiology, University of Split, Split, Croatia

**Keywords:** Strength training, Aging, Physical fitness, Total cholesterol, High density lipoprotein, Fasting glucose

## Abstract

**Background:**

Resistance training is recognized as an effective way to improve muscle strength, metabolic health, and functional fitness in older adults. However, there is little agreement on the ideal number of resistance training sessions per week. It is important to clarify the comparative effects of various training frequencies, especially with accessible low-load methods like elastic bands, to create practical and effective exercise recommendations for this population.

**Methods:**

This study compared the effects of two (2 sessions/week;2SW) versus three (3 sessions/week;3SW) low-load elastic band resistance training sessions over 12 weeks on metabolic biomarkers and functional fitness in elderly women. Eighty-two women (75.1 ± 8.6 years) were randomly assigned to 2SW (*n* = 41) or 3SW (*n* = 41) groups. In this single blind randomized controlled trial, both groups performed equal training volume per session:2 sets of 12–15 repetitions at 40–50% one-repetition maximum using elastic bands. Metabolic biomarkers (fasting glucose, triglycerides, total cholesterol, HDL, LDL) and functional fitness (Chair Sit-and-Reach, 2-min Step Test, 30-s Chair Stand, 30-s Arm Curl, Back Scratch, 2.4-meter Up-and-Go) in addition with handgrip strength were assessed before and after the intervention. Adherence exceeded 90% in both groups.

**Results:**

Baseline measures were similar between groups (*p* > 0.05). Both groups improved significantly in 30-s Chair Stand, 2-min Step test, Arm Curl, Chair Sit-and-Reach, Back Scratch, and Handgrip Strength. 2.4-meter Up-and-Go test improved only in the 2SW group. The 3SW group showed greater increases in handgrip strength (+ 28% vs. +7%) and HDL cholesterol (+ 9% vs. +1%), while total cholesterol and LDL reductions were comparable between groups (*p*< 0.05). No significant changes occurred in fasting glucose or triglycerides.

**Conclusion:**

Findings indicate that both two and three low-load elastic band resistance training sessions per week significantly enhance handgrip strength, functional fitness and several metabolic biomarkers profile, with three sessions providing additional benefits in muscle strength and HDL cholesterol.

## Introduction

Aging is a natural process associated with decline in muscle volume and strength [1], accompanied with other functional fitness impairments [2] and various negative changes in health status including metabolic biomarkers [3]. This downward cycle leads to functional decline [4], with detrimental changes in serum blood glucose (GLU) and lipid levels that have been shown to increase cardiovascular disease risk and premature death substantially, especially in women [5, 6]. Emerging evidence from scientific research indicates that strength enhancement through targeted resistance training is a fundamental mechanism for inducing beneficial physiological and functional adaptations in older adults. Resistance training is pivotal in attenuating age-related declines in muscular strength and overall functional performance as well as metabolic biomarkers thereby facilitating improved health outcomes and elderly quality of life [7].

Elastic band resistance training has recently gained popularity as a valid alternative to traditional free weights and machines [8–10]. Portable and cost effective, this method is valued for its versatility, accessibility for various age groups, adaptability to different clinical environments [11, 12] and high adherence rates [13]. Benefits are particularly pronounced for the elderly, including those new to strength training, with significant short-term health improvements reported [14]. Although higher training intensities correlate with better health outcomes, low-load elastic band exercises (below 65% of one-repetition maximum) are less likely to cause injury [15, 16]. Furthermore, low-load elastic band exercises consistently improve functional fitness and metabolic health in older adults [17]. Thus, low-load elastic band training is a safe, effective, and accessible option for enhancing strength and health in this population.

The magnitude of responses induced by resistance training in older adults is influenced by the manipulation of variables that compose training programs, such as exercises selection and order, number of sets and repetitions, contraction velocity, rest intervals and notably training frequency [18, 19]. Among these variables, the training frequency may significantly influence the design of resistance training (RT) programs [20]. It is important to note that if frequency is manipulated alongside volume (i.e., total weekly sets differ between groups), this may confound the isolated effects of frequency. While such a design reflects practical training scenarios, it complicates distinguishing whether observed outcomes result from frequency itself or differences in total training volume. Older adults generally need longer recovery times between training sessions compared to younger individuals because age-related physiological changes impact muscle repair and fatigue management [21]. Sufficient recovery between training sessions can mitigate fatigue buildup, facilitate the healing of exercise-induced microtraumas, and facilitate optimal resistance training adaptations in older adults [22]. Therefore, expert guidelines generally advise to implement 2 to 3 sessions per week of comprehensive whole-body RT programs for this population [23]. However, empirical evidence directly comparing the effects of two versus three weekly resistance training sessions remains scarce and inconclusive.

A recent study by Dos Santos et al. [24] demonstrated that a 12-week resistance training program conducted twice weekly was as effective as a thrice-weekly regimen in enhancing muscular strength among sarcopenic elderly women. Similarly, Pina et al. [25] reported comparable improvements in muscular strength across training frequencies with two or three sessions, per week when the total number of sets was matched. In addition, increased training frequency was associated with a more substantial reduction in body fat among elderly women. Finally, Nascimento et al. [26] reported no differences between groups (2 vs. 3 sessions per week) in strength, fasting glucose, triglycerides, total and HDL cholesterol after 12 weeks of resistance training in elderly woman. In contrast, Farinatti et al. [27] investigated the impact of varying training frequencies on muscle strength and functional performance in physically active women aged over 60 years. Their findings indicated that higher-frequency training protocols yielded greater enhancements in both strength and functional capacity. Similarly, a subsequent study involving 127 mixed-gender older adults (aged 60–85 years) reported comparable outcomes, demonstrating dose-dependent improvements in muscle mass, strength, and physical function as a consequence of limited-load resistance training conducted at higher frequencies [28].

Consequently, there is still a need for studies focused on the effects of varying resistance training (RT) frequencies on muscular strength and other health indicators, including metabolic biomarkers, particularly among older women. Examining the varying impacts of different frequencies is essential to determine if higher session frequencies offer significant benefits or if a lower frequency can produce comparable results. Understanding this will help professionals customize efficient training programs, considering factors such as time constraints, recovery ability, and adherence among older adults. Additionally, as far as we are aware, no research has yet investigated the fitness and health effects of low-load elastic band resistance training frequency in this specific cohort. Therefore, this study intends to evaluate the effects of two vs. three low load elastic band resistance training sessions per week on strength, functional fitness and metabolic biomarkers in elderly women. We hypothesized that greater training frequency would lead to greater improvements in functional fitness and metabolic biomarkers in a group of healthy women over the age of 65 years.

## Materials and methods

### Study design

This was a assessor-blinded, randomized controlled trial conducted across three geriatric centers in Vojvodina, Serbia (Zrenjanin, Subotica, Novi Sad). While the coaches supervising the interventions were aware of group allocations due to the difference in training frequency, all outcome assessments—including metabolic biomarker measurements and functional fitness testing—were performed by personnel blinded to group assignment and unaffiliated with training supervision. This ensured unbiased measurement of study outcomes. Block randomization by site, done by a researcher not involved in the study and unaware of the aims, was performed to ensure balanced group allocation within each center. Standardized training protocols and coaching procedures were implemented across centers to minimize heterogeneity. Nevertheless, differences in facilities, coaching styles, or participant demographics may have contributed to variability in outcomes. Due to sample size constraints, formal exploratory center-level analyses were not conducted. Within the week following medical screening, participants underwent baseline testing, including blood sampling, anthropometric measurements (height and body mass measured with Tanita model BF-350), and functional fitness assessments [29], with addition of handgrip strength. Testing was conducted in the early mornings (7:30 a.m.) under standardized conditions. Participants were familiarized with testing procedures at least 48 h prior to testing. Testing procedures, equipment, and personnel remained consistent across assessments, with all measurements performed in controlled room conditions (temperature around 22 °C, humidity around 50%). All tests’ procedures were conducted with qualified personnel unaffiliated with training supervision. Following the 12-week training regimen, participants underwent post-intervention testing identical to baseline assessments.

The training program lasted 12 weeks and utilized elastic bands, with the training protocol standardized across centers. A potential limitation is that participants were aware of their assigned training frequency, which may have influenced motivation and effort during effort-dependent functional tests. However, both groups were equally informed about the potential benefits of the intervention and encouraged to give maximal effort, aiming to minimize differential expectation effects. Nevertheless, such influences are difficult to fully control in exercise interventions without participant blinding, and some observed improvements may partially reflect motivational factors. This should be considered when interpreting the functional performance results. Coaches supervising the interventions, two in each geriatric center, due to the nature of session frequency, were not blinded to group allocation. However, to minimize bias, they followed standardized protocols and neutral coaching approaches across both groups. Participants and institutional supervisors were instructed to maintain their usual behavior and diet throughout the study.

### Participants

**Fig. 1:**
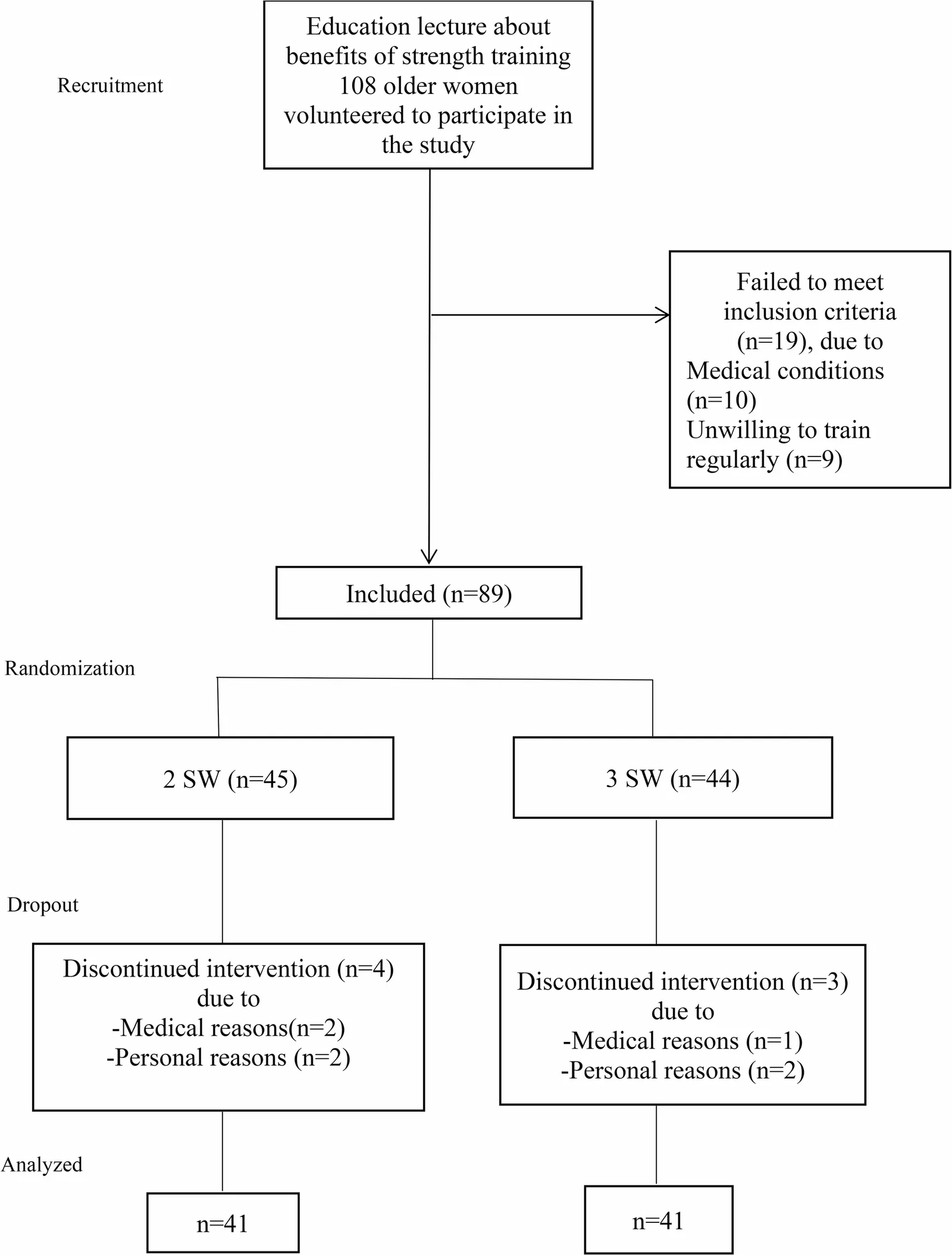
Flow diagram of study participants

Sample size estimation was performed using G*Power (version 3.1.9.3, Düsseldorf, Germany). An effect size of 0.20, an α level of 0.05, and a power (1 - β) of 0.80 suggested that a minimum of 76 volunteers (37 subjects per group) would need to be included. An effect size of f = 0.20 was selected for the power analysis, representing a small to moderate effect consistent with prior resistance training studies in older adults demonstrating similar magnitudes of change in muscle strength and functional fitness outcomes [26]. Initially, a total of 108 female residents expressing interest in the study were recruited following an educational lecture highlighting the benefits of strength training. Participants underwent medical screening for suitability to perform resistance training, conducted over two days in each center. Exclusion criteria included orthopedic, neurologic, rheumatologic, or cardiovascular disorders, as well as participation in organized physical activity within the previous two years. Nineteen women were not eligible for the study. Thus, 89 women met the inclusion criteria, signed informed consent and were randomly allocated into two groups. For further inclusion in data analysis subjects had to complete 85% of training sessions. Finaly, 82 subjects completed the whole experiment (Fig. 1). Three participants discontinued for medical reasons unrelated to the intervention, and four withdrew for personal reasons.

### Measures

Participants received clear instructions to refrain from engaging in physical activity and consuming beverages containing alcohol or caffeine for 24 h and 3 days prior to testing, respectively. Blood samples were drawn by a qualified medical professional from the superficial veins of the upper arm after an overnight fast. The collected blood was stored in serum separator tubes and subsequently processed by centrifuging the samples for 10 min at 3000 rpm to separate the serum. The serum concentrations of glucose (GLU), total cholesterol (TC), high-density lipoprotein cholesterol (HDL), low-density lipoprotein cholesterol (LDL), and triglycerides (TG) were determined in a certified laboratory at the Novi Sad city clinic. All biochemical assessments were performed using an automated analyzer system (MINDRAY BS 800; Bio-Medical Electronics Co., Shenzhen, China), following standard protocols. The Friedewald equation was applied to estimate LDL cholesterol levels [30].

The assessment employed the Senior Fitness Test battery (SFB), complemented by a handgrip strength assessment. The SFB is a six-item field test battery, specifically developed and validated to evaluate key aspects of functional fitness in older adults, including strength, flexibility, dynamic balance, and cardiovascular endurance [29]. The tests included were: Handgrip Strength (kg), 30-second Chair Stand (repetitions), 30-second Arm Curl (repetitions), Chair Sit-and-Reach (centimeters), Back Scratch (centimeters), 2.4-meter Up-and-Go (seconds), and a 2-minute Step Test (count of knee lifts within the time frame). The handgrip strength measurement was incorporated as a general health indicator [31] and was performed following the protocol outlined by Fess [32]. The entire SFB was administered according to the guidelines provided by Rikli and Jones [29]. Prior to testing, participants completed a warm-up lasting between 5 and 10 min. All tests, except for the 2-minute step test, were performed twice, with the highest score recorded. For the isometric handgrip assessment, the maximum combined strength (left + right) from three trials, separated by one-minute rest periods, was used for analysis. The reliability of both the SFB and handgrip test was evaluated through a test-retest procedure in a pilot sample of 20 randomly selected individuals from Novi Sad. Results showed intraclass correlation coefficients ranging from 0.87 to 0.92 across all measures, demonstrating excellent reproducibility of the selected functional assessments.

### Training protocol

Training interventions were performed using light-intensity elastic bands (Thera-Bands, The Hygenic Corporation, Akron, OH, USA). Patients were organized in groups, with no more than 10 in one group. Strength training exercises consisted of 12 chair-based exercises, and included muscle groups from hip/knee/shoulder/trunk (Table 1).

**Table 1:** Strength training exercises. RPE-O=rating of perceived exertion OMNI-scale

Exercise	Sets	Reps	Cadence	Rest	RPE-O
Seated EB knee extension	2	12–15	3 s + 3 s	60 s	4–5
Seated EB knee flexion	2	12–15	3 s + 3 s	60 s	4–5
Seated EB horizontal pull	2	12–15	3 s + 3 s	60 s	4–5
Seated EB horizontal push	2	12–15	3 s + 3 s	60 s	4–5
Seated EB hip flexion	2	12–15	3 s + 3 s	60 s	4–5
Seated EB trunk rotation- oblique	2	12–15	3 s + 3 s	60 s	4–5
Seated EB trunk flexion	2	12–15	3 s + 3 s	60 s	4–5
Seated EB high pull	2	12–15	3 s + 3 s	60 s	4–5
Seated EB elbow flexion	2	12–15	3 s + 3 s	60 s	4–5
Seated EB overhead elbow extension	2	12–15	3 s + 3 s	60 s	4–5
Seated EB hip lift and abduction	2	12–15	3 s + 3 s	60 s	4–5
Standing EB kickback	2	12–15	3 s + 3 s	60 s	4–5

All training sessions were supervised by two qualified and experienced strength and conditioning coaches for every center. Each training session consisted of 3 different parts. Sessions started with a warm-up procedure lasting 10 min using mobility exercises. Structured strength training was a main part of the training session. The cool-down procedure consisted of stretching exercises, each one per muscle group, with 2 × 20–30 s of static stretching. Each strength training session consisted of 2 sets of 12–15 reps, with 60 s of rest between each set, two and three times per week (depending on the group), with at least 48 h between each training session. Exercises were performed by 3 s of eccentric and 3 s of concentric contraction, without pause between regime and repetitions. Training intensity ranged from 4 to 5 using OMNI resistance for active muscle scale, which is equal to 40% and 50% of 1RM. The idea was to maintain internal load during the study in order to follow the concept of low-load training effects on selected measures. Since the study lasted 12 weeks in duration, participants increased the strength levels during the intervention, and to preserve training effects stagnation (when ratings went below 4), we increased the exercise intensity by changing the length of the hand grip or by using different band colors. Different order of exercises was applied each week, trying to maintain participants’ motivation. Qualified instructors recorded the attendance of each participant.

### Statistical analysis

The descriptive data are presented as means ± standard deviation (SD). Since the Kolmogorov-Smirnov test indicated that all parameters followed a normal distribution, parametric statistical tests were utilized. Baseline differences between groups were examined using a two-sample Student’s t-test. To evaluate changes from pre- to post-intervention and assess differences between groups, a two-way repeated measures ANOVA with a 2 (groups) x 2 (time points) design was conducted. When the F-test was significant, the LSD post hoc analysis was performed to pinpoint specific group differences. Given the limited number of groups and comparisons, LSD was chosen for its sensitivity in detecting meaningful differences without undue conservatism that may increase Type II errors. This approach is supported by methodological literature indicating LSD’s appropriateness in designs with few comparisons and when the overall ANOVA is significant [33]. We explicitly applied LSD to identify pre- to post-intervention changes within each group and to clarify the direction of significant interactions. The effect size of the mean differences was quantified using Cohen’s d, calculated by subtracting the means and dividing the result by the pooled standard deviation. According to Cohen’s thresholds, a Cohen’s d of 0.00–0.19 indicates a trivial effect, 0.20–0.49 a small effect, 0.50–0.79 a moderate effect, and ≥ 0.80 a large effect [33]. The percentage change for each variable was determined using the formula: ([post-value / pre-value] − 1). Statistical significance was set at a two-tailed p-value ≤ 0.05. Data analysis was performed with IBM SPSS Statistics version 22 (IBM Corp., 2013, IBM SPSS Statistics for Windows, Version 22.0, Armonk, NY). Because the study involved a limited number of pre-planned comparisons, no corrections for multiple comparisons were applied. This approach is consistent with standard practice in hypothesis-driven research where the number of comparisons is small and defined a priori. Reporting effect sizes and confidence intervals alongside p-values allowed for a more nuanced interpretation of the results while balancing Type I error risk and statistical power.

## Results

For determining the effects of strength training frequency performed 2 and 3 times per week on metabolic biomarkers and functional fitness in elderly, data were obtained from a sample of 82 elderly women. Their basic descriptive statistics are presented in Table 2, and the results are shown as mean ± standard deviation. Participants included in the analysis attended 90% of the training sessions. Furthermore, no injury or illness were recorded in any participant during the experimental treatment. At the initial measurement, no statistically significant differences were found between 2SW and 3SW (Table 2) (*p* > 0.05).

**Table 2:** Physical characteristics of participants

Variable	2SW Group	3SW Group	Total
	*n* = 41	*n* = 41	*n* = 82
Age	74.5 ± 8.2	75.7 ± 8.9	75.1 ± 8.5
Body height (cm)	160.2 ± 5.6	162.4 ± 6.3	160.0 ± 6.0
Body weight (kg)	70.8 ± 12.3	72.3 ± 11.6	71.6 ± 11.9

Statistically significant differences were found for total cholesterol (d = 1.19 and 0.72 ) and LDL (d = 1.02 and 0.92) between the initial and final measurement in 2SW and 3SW, respectively, as well as statistically significant differences for HDL (d = 0.93) for 3SW (Table 3). Furthermore, HDL showed a statistically significant interaction of time*group (*p* < 0.05).

**Table 3:** Results of the two-way ANOVA for biochemical analysis for Group 1 and Group 2. Values are presented as mean±standard deviation. HDL-high density lipoprotein; LDL-low density lipoprotein; *significant difference for pre/posttest at *p* < 0.05; † significant difference between Group 1 and Group 2 at *p* < 0.05

Variable			2SW Group (*n* = 41)			Change%			3SW Group (*n* = 41)			Change%
	Pretest	CI 95%		Posttest	CI 95%		Pretest	CI 95%		Posttest	CI 95%	
Glucose (mmol/l)	5.53 ± 1.40	[5.09, 5.98]		5.29 ± 1.55	[4.80, 5.80]	-4.	6.23 ± 1.91	[5.62, 6.83]		5.89 ± 1.82	[5.31, 6.42]	-5.
Total cholesterol (mmol/l)	6.31 ± 1.53	[5.82, 6.79]		5.73 ± 1.45*	[5.28, 6.19 ]	-9	5.83 ± 0.96	[5.53, 6.14]		5.50 ± 0.91*	[5.21, 5.79]	-6
HDL (mmol/l)	1.34 ± 0.34	[1.23, 1.45 ]		1.35 ± 0.36	[1.24, 1.45]	1	1.29 ± 0.40	[1.16, 1.42]		1.40 ± 0.35*†	[1.29, 1.51]	9
LDL (mmol/l)	4.21 ± 1.40	[3.76, 4.65]		3.72 ± 1.21*	[3.34, 4.10]	-11	3.76 ± 0.94	[3.46, 4.06]		3.36 ± 0.78*	[3.11, 3.60]	-11
Triglycerides (mmol/l)	1.57 ± 0.60	[1.38, 1.76 ]		1.58 ± 0.55	[1.40, 1.75]	1	1.75 ± 0.87	[1.48, 2.02]		1.74 ± 0.78	[1.38, 1.76]	-1

Both groups showed a statistically significant difference between the initial and final measurement for the following variables: 30-second chair stand test (d = 1.60 and 1.32), chair sit and reach (d = 0.71 and 0.92), 30-second arm curl test (d = 1.17 and 2.10), 2-minute step test (d = 1.93 and 1.45) back scratch test (d = 0.99 and 1.00), and hand grip test (d = 0.99 and 3.20) for 2SW and 3SW respectively; a significant difference between the initial and final measurement for 2.4 m up and go test was found only in 2SW (d = 1.93). Significant interaction of time*group was found for the hand grip test (*p* < 0.05) (Table 4).

**Table 4:** Results of the two-way ANOVA for physical form parameters in Group 1 and Group 2. Values are presented as mean±standard deviation; *significant difference for pre/post test at p < 0.05; † significant difference between Group 1 and Group 2 at p < 0.05

Variable		2SW Group (*n* = 41)			Change%			3SW Group (*n* = 41)			Change%
	Pretest	CI 95%	Posttest	CI 95%		Pretest	CI 95%		Posttest	CI 95%	
30-second chair stand (n/30s)	14.59 ± 7.20	[12.32, 16.85]	16.78 ± 7.30*	[14.48, 19.09]	15	10.15 ± 4.78	[8.64, 11.65]		11.59 ± 3.90*	[10.35, 12.82]	14
30-second arm curl (n/30s)	20.27 ± 8.15	[17.70, 22.84]	22.39 ± 8.35*	[19.75, 25.03]	10	14.93 ± 4.77	[13.42, 16.43]		18.34 ± 5.12*	[16.72, 19.96]	23
2-minute step test (n/2min)	85.24 ± 36.0	[73.88, 96.60]	100.27 ± 40.75*	[87.40, 113.13]	18	70.95 ± 29.1	[61.77, 80.13]		82.54 ± 25.70*	[74.43, 90.64]	16
Chair sit and reach (cm)	-1.35 ± 10.48	[-4.66, 1.96]	1.56 ± 9.17*	[-1.50, 4.63]	N/A	-0.68 ± 8.61	[-3.39, 2.04]		1.78 ± 5.37*	[0.09, 3.48]	N/A
Back scratch test (cm)	-10.15 ± 13.87	[14.53, -5.77]	-6.87 ± 12.37*	[10.77, 2.96]	-32	-12.57 ± 12.7	[16.41, -8.73]		-9.28 ± 10.14*	[12.48,-6.08]	-26
2.4 m Up-and-Go Up (s)	9.75 ± 7.40	[7.41, 12.09 ]	8.60 ± 7.0*	[6.38, 10.80]	-12	9.22 ± 4.80	[7.70, 10.73]		8.68 ± 3.80	[7.48, 9.87]	-6
Hand grip test (kg)	39.61 ± 12.11	[35.78, 43.43]	42.49 ± 12.54 *	[38.53, 46.45 ]	7	28.24 ± 14.43	[23.69, 32.80]		36.05 ± 15.62*†	[31.12, 40.98]	28

N/A- Non applicable. When baseline values approach zero or are negative, proportional change cannot be meaningfully expressed.

## Discussion

The present study aimed to evaluate the effect of 2 vs. 3 sessions per week of low-load elastic band resistance training on muscular strength, functional fitness and metabolic biomarkers in older women. The 3SW group showed significantly larger improvements in handgrip strength and HDL cholesterol than 2SW. There were significant decreases in both groups in total cholesterol and LDL, with an increase in HDL cholesterol observed in the 3SW group only. Both groups achieved significant improvements in most functional fitness measures, except for the 2,4 m up and go test, which saw enhancements only in the 2SW group. Both training programs significantly improved functional fitness and several metabolic biomarkers in elderly women, with the 3SW group achieved greater benefits in strength and HDL. Thus, our hypothesis was partially supported: three sessions per week produced greater improvements in handgrip strength and HDL cholesterol, but the anticipated advantage in broader functional fitness outcomes was not observed.

Before considering these findings in relation to prior literature, it is worth briefly addressing the baseline profile of the two groups. Although block randomization by site was performed by a researcher blinded to the study aims, and no baseline differences reached statistical significance, groups showed some numerical variation at baseline for several outcomes. This is consistent with chance imbalance, which can occur even with correctly implemented randomization when sample sizes are moderate and outcome variability is high, and does not reflect a flaw in the allocation process.

The strength results in our study are partly in line with some previously published data revealing that higher training frequency leads to greater strength gains. Farinatti et al. [27] reported significantly larger strength improvements in women over 60 years training three times per week compared to one and two training per week. Observed strength increases ranged from 40% for calf raise up to 69,9% for dumbbell curl; longer study duration (16 weeks) and higher loads (70% 1RM) likely explains greater gains than observed in current study. Similarly, in a recently published systematic review, Li et al. [28] reported that frequency of three times per week produced superior handgrip strength improvements in comparison to lower frequency in older sarcopenic adults. Conversely, several studies showed no significant strength differences with increased frequency. DiFrancisco-Donoghue et al. [34] found that older adults who trained twice per week showed slightly greater but non-significant strength improvements compared to those training once per week. It should be noted, however, that the small sample size (7 women and 11 men) is likely responsible for the lack of significant results. Taaffe et al. [35] reported comparable strength improvements across one, two, or three sessions per week using 3 sets of 80% 1RM for 24 weeks. More recently, dos Santos et al. [24] compared 12 weeks of resistance training twice versus thrice weekly in sarcopenic older women and found significant strength improvements in both groups, with no statistical difference between them. Notably, strength gains in some exercises were non-significantly greater in the thrice-weekly group, suggesting that higher volume, such as adding an extra set—as implemented in our study—may amplify strength improvements and highlight differences between frequencies. In a well-conducted study, Pina et al. [25] examined the effects of equi-volume, two versus three resistance training sessions per week over a period of 12 weeks (2 × 3 sets versus 3 × 2 sets, with 10–15 repetitions per set) in older adults. Both groups exhibited significant increases in bench press (+ 11.8% and + 11.9%) and knee extension (+ 17.4% and + 10.8%) one-repetition maximum (1RM), showing no significant differences between groups. Current study design differed in training volumes between groups (4 sets for the 2SW group versus 6 sets for the 3SW group), which likely accounts for the significant strength differences reported. Interestingly, our protocol involving two sets of 12–15 repetitions at a low load (40–50% of 1RM) across 12 exercises proved effective in achieving substantial strength gains with just twice-weekly training—a volume around 50% lower than utilized by Pina et al. [25]. These findings may be important for frail older women who struggle to keep up with consistent exercise routines.

The results for functional performance showed improvements in all tests except the 2.4 m up and go for the 3SW group, with no significant differences between training frequencies. Interestingly, the 2.4-meter Up-and-Go test improved significantly only in the 2SW group. This counterintuitive finding may reflect accumulated neuromuscular fatigue in the higher-frequency (3SW) group. The 2.4-meter Up-and-Go test requires rapid, coordinated multidirectional movements and may be particularly sensitive to fatigue-induced impairments [36]. The increased training volume and reduced recovery periods in the 3SW protocol likely elevated residual fatigue, transiently compromising performance on this agility-specific assessment despite overall strength and functional gains.

Functional outcomes appeared largely unaffected by frequency, which aligns with limited previous findings. Farinatti et al. [27] found that chair sit and stand and gait speed tests improved significantly after 16 weeks of training (10 exercises, 1 set of 10 reps at 70% 1RM) in groups training twice or thrice weekly, with greater improvements than once-weekly training but no difference between two- and three-session groups, consistent with our findings. Turpela et al. [36] reported meaningful improvements in four functional tests (forward and backward walk, timed up and go, loaded 10-stair climb) after 6 months of training twice or three times per week, with no frequency differences despite training intensities of 70–90% 1RM.Similarly, Li et al. [28] found that training more than three times weekly led to greater improvements in sit-to-stand and 6-minute walk tests compared to lower frequency programs after six months. Taken together, these findings suggest that two or three weekly strength training sessions yield similar functional performance benefits in older women, regardless of training intensity (from 40% 1RM in our study to 90% in Turpela et al. [37]. Participation in strength training per se is critical for enhancing functional capacity, while the magnitude of strength gains seems to have little direct effect on functional improvements. A methodological factor possibly influencing our results is test selection. We used an isometric handgrip test, while all training involved dynamic exercises. This non-specific measure may decrease sensitivity to training-induced changes and attenuate the relationship between strength and functional results as observed in similar studies [37].

Recent studies increasingly show that strength training positively affects various metabolic markers. For example, Ibáñez et al. [38] found improved cardiovascular risk factors in obese women following resistance training. Similarly, Conceição et al. [39] demonstrated reduced metabolic syndrome risk in healthy postmenopausal women after 16 weeks of resistance training. Fahlman et al. [40] reported favorable plasma lipoprotein changes in elderly women following endurance and resistance training. Moreover, low-intensity strength training at approximately 50% of 1RM using elastic bands, similar to our study design, has been shown to improve metabolic markers [17]. Metabolic adaptations appear highly dependent on program design, especially load, volume, and intensity [41]. However, research on how training frequency alone affects metabolic biomarkers is limited and inconclusive. Contrary to our findings, Ihalainen et al. [42] showed that high-intensity resistance training (70–90% 1RM), performed once to three times weekly, increased HDL cholesterol significantly in older adults, with no greater benefits from higher frequency. Their group training thrice weekly was the only group to show significant LDL cholesterol reduction, differing from our results. This discrepancy may stem from baseline LDL differences, as our twice-weekly group started with higher LDL, possibly explaining their significant decrease. Likewise, Nascimento et al. [26] found reductions in fasting glucose and triglycerides without differences between two to three weekly sessions, and LDL reductions were consistent with our findings. In older women, their 12-week program consisted of one set of 10–15 repetitions per session. A 24-week study by Pina et al. [43] also found no differences between two and three weekly sessions in blood glucose and HDL improvements, with no significant differences in total cholesterol, LDL, or triglycerides. Taken together, these studies suggest that different training frequencies have similar clinical effects on metabolic biomarkers in older females. While some biomarkers show significant changes pre- to post-intervention across studies, not all do. Our findings concur, with total cholesterol and LDL improving significantly, but not fasting glucose or triglycerides. Notably, the three-times-weekly group in our study improved HDL cholesterol significantly more than the two-times group, suggesting higher frequency may enhance specific metabolic markers. The notable improvement in HDL cholesterol observed with three sessions of low-load resistance training per week in older women is likely attributed to several factors, including a higher volume of exercise [44], enhanced mitochondrial and metabolic adaptations in muscle [39], increased anti-inflammatory and hormonal responses [42], and improved enzyme activity that regulates HDL metabolism [41]. These results reinforce the idea that incorporating higher-frequency resistance training could be beneficial for optimizing cardio-metabolic health in aging individuals.

There are several limitations to this investigation. Initially, the absence of monitoring physical activity and sedentary habits during the study restricts our ability to assess whether these factors may have influenced the observed changes in the variables measured over time. Additionally, dietary intake was not standardized or regulated, which could have introduced confounding effects by impacting metabolic biomarkers independently. Furthermore, the absence of a control group makes it difficult to determine the true effect size of the intervention. Nevertheless, prior research suggests that low-intensity resistance training using elastic bands can serve as an effective strategy for improving the variables examined in this study [17]. Lastly, a notable limitation is that training frequency was manipulated alongside total weekly training volume, with the three-session group completing 50% more sets per week than the two-session group. This confound restricts the ability to isolate the effects of frequency from volume, as differences in outcomes may be due to either factor or their combination. Although this design reflects practical training scenarios, future research should consider volume-matched protocols to clarify frequency-specific effects more definitively.

## Conclusions

Engaging in two to three low-load strength training sessions per week yields comparable improvements in functional fitness and nearly all measured metabolic biomarkers. Additionally, performing strength training more frequently may lead to greater enhancements in muscle strength and HDL cholesterol levels, following a 12-week intervention.

## Data Availability

The dataset used and analyzed during the current study is available from the corresponding author upon reasonable request.

## Abbreviations

1RM: One-Repetition Maximum
2SW: Two Sessions per Week
3SW: Three Sessions per Week
ANOVA: Analysis of Variance
BF: Body Fat
CI: Confidence Interval
EB: Elastic Band
GLU: Fasting Glucose
HDL: High-Density Lipoprotein Cholesterol
LDL: Low-Density Lipoprotein Cholesterol
OMNI: OMNI Resistance Scale (Rating of Perceived Exertion)
RT: Resistance Training
SD: Standard Deviation
SFB: Senior Fitness Test Battery
TG: Triglycerides
